# 5-alpha-reductase and the development of the human prostate

**DOI:** 10.4103/0970-1591.42610

**Published:** 2008

**Authors:** C. Radmayr, A. Lunacek, C. Schwentner, J. Oswald, H. Klocker, G. Bartsch

**Affiliations:** Department of Pediatric Urology, Medical University Innsbruck, Austria; 1 Department of Urology, Medical University Innsbruck, Austria

**Keywords:** 5-alpha-reductase, fetal development, human prostate

## Abstract

During the 10^th^ week of gestation human prostate development is about to start. Androgens are the crucial factors to stimulate the initial interactions between the epithelium and mesenchyme. One of the key events in androgen metabolism is the transformation of circulating testosterone to 5α-dihydrotestosterone (DHT) by tissue-linked 5α-reductase. Both, the formation of a male phenotype and the androgen-mediated growth of the prostate are mediated by DHT. To date the function of 5α-reductase 1 (5αR1) still remains unclear whereas 5α-reductase 2 (5αR2) is supposed to be the predominant isoenzyme in human accessory sex tissue. Only little data are available on the detection, distribution, and effects of both isoenzymes during fetal life and infancy. Recently, immunohistochemical investigations of serial sections from fetuses and infants using specific antibodies directed against 5αR1 and 5αR2 seem to shed light on that issue. Moreover, the detection of downstream products of androgen synthesis using RT-PCR analyses for 17-β hydroxysteroid dehydrogenase Type 2 (17 βHSD 2), 17 βHSD Type 3 and 17 βHSD Type 7 adds to discovering the molecular biological background. New studies confirm that both isoenzymes are present throughout fetal development. On the transcriptional level RT-PCR for 5αR1 and 5αR2 certifies these findings. 17 βHSD 2, 3 and 7 representing the most relevant enzymatic downstream products of cellular androgen synthesis were revealed by RT-PCR as well. Current studies discovered the expression and distribution of both 5α-reductase isoenzymes as well as the potential contribution of 5αR1 during fetal human prostate development.

## INTRODUCTION

This review addresses the issues of the distinct role of 5-alpha-reductases during fetal development. There are still a lot of controversies and lack of data with regard to the expression and distribution of the different isoforms as well as their influence and meaning for different target tissues during organogenesis as well as during the development of benign or malign disorders. There are a lot of more questions than data although 5-alpha-reductase inhibitors are widely used therapeutically in the treatment of different prostatic diseases and have been proven to be efficient in randomized clinical trials in the treatment of benign prostatic hyperplasia.[1]

## EVIDENCE

It is certain that for fetal prostate development androgens are essential and represent the key factors. 5αR1 and 5αR2 enzymatically transform testosterone to the more potent androgen 5α-dihydrotestosterone (DHT). These two specifically bind to the cell membrane located androgen receptor, a nuclear steroid receptor, being widely abundant in developing and adult tissue. Phenotype development and target tissue priming is mediated by tissue-linked reductases. The function of 5-alpha-reductase 1 (5αR1) regarding its effects on urogenital sinus development still remains unclear while 5-alpha-reductase 2 (5αR2) is considered predominant in human accessory sex tissue and is responsible for prostate and male external genitalia development.[2,3] The same is true for downstream products of DHT-action, namely 17β-hydroxysteroid-dehydrogenase 2 (17βHSD 2), 3 (17 βHSD 3), and 7 (17 βHSD 7).[2] They act as steroidogenic enzymes regulating hormone homeostasis in target organs.

Meanwhile it has been proven that abnormalities in androgen metabolism, reductase activity and the androgen receptor can result in a broad spectrum of disturbances in sex development.[3]

Realizing the physiological role of 5αR1 and 5αR2 it is necessary to expose their expressional time itinerary and allocation within the developing prostate. Therefore investigating the involvement of both isoforms in branching and budding processes in the fetal prostate is mandatory.[4] Research focusing on the basics of prostate cancer and benign prostatic disorders already emphasized the pathophysiological importance of both enzymes.[4] As a final point, the central role of 5αR1 in human genital development should be revisited.

## INFLUENCE OF 5αR1 AND 5αR2 ON THE FETAL DEVELOPMENT OF THE PROSTATE

Recent data confirm that not only 5αR2 but also 5αR1 may contribute to fetal development of the human prostate although there is still discussion about the relevance of 5αR1 in prostate development; still some advocate no involvement whereas others already admit its biological influence.[5]

It is a well-recognized fact that prostate development depends on the action of both, testosterone and DHT.[4] 5αR1 and 5αR2 are fundamental in androgen physiology; testosterone is transformed into the more compelling androgen DHT by 5-Δ[4] ketosteroid alpha reductase (5αR) augmenting androgen effects in target tissues.

On the genomic level 5αR1 is located on Chromosome 5p15 and 5αR2 on Chromosome 2p23, respectively.[6] Testosterone and DHT act through the androgen receptor, a transcription factor belonging to the superfamily of steroid receptors. DHT joins with a superior affinity to the receptor than testosterone.[7] DHT action affects the urogenital sinus and external genitalia being indispensable for both, male phenotype configuration and androgen-mediated development of the prostate.[4]

**Figure 1:**
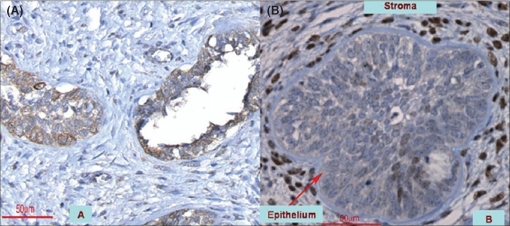
(A and B) Expression of 5αR1 and 5αR2 in the fetal prostate (magnification 400×): (A) Within the epithelium 5αR1 is primarily found while only few scattered cells are identified in the stroma (21^st^ week); (B) 5αR2 exhibits a much weaker staining intensity in the epithelial compartment whereas it is significantly more abundant in stromal cells (27^th^ week)

Recent publications discovered the topography and molecular expression of 5αR1 and 5αR2 within the developing prostate [Figure 1A, B]. Moreover, downstream products of the androgen response participating in the metabolization of DHT like 17 βHSD were identified as well.[8] In this study the use of RT-PCR confirmed the transcription and tissue availability of 5αR1 and 5αR2 in fetal and newborn prostates. Furthermore, the expression of 17 βHSD 2, 3, and 7 was notable proving a functional androgen pathway. 5αR1- and 5αR2-transcription exposed a developmental reliant time course: peak mRNA-production was perceived at the edge of the second and third trimester paralleled with a rise of testosterone levels. Subsequently, 5αR-expression returned to the point of early gestation remaining at that level postnatally. Consequently, except from mid-gestation 5αR-production appeared to be comparable during all stages. Kinetics of 17 βHSD 2, 3, and 7 illustrated different patterns: transcription of 17 βHSD 2 and 7 was highest during the sixth month, being reinforced again in infancy. Unlike those, excessive Type 3 expression was only seen postnatally.

5αR1 and 5αR2 are notable within epithelium and stroma starting as early as from the ninth week of gestation. Testosterone production leads 5αR-expression by one week, being a potential trigger for this event.[4,9] Both enzymes are in attendance in both compartments although with varying density. Fetal and infant specimens also exposed their existence on the transcriptional level. Budding and branching areas particularly expressed 5αR1 implying involvement in the epithelial-mesenchymal interactions. Consequently, 5αR1 may control integrin and protease production in spreading prostatic epithelial cells.[4] On the other hand there are some authors denying the existence of 5αR1 in the fetal prostate. Some conclude that 5αR1-expression begins at around birth and 5αR2 is fundamental in male genital development.[10] But one has to point out that they studied genital skin of fetuses and neonates while the prostate was analyzed postnatally only. Moreover, females were also included in the study, limiting their conclusions. 5αR1 was present in the normal prostate of adolescents and adults only. In prostate cancer and hyperplasia they only noted 5αR2. Meanwhile the presence of 5αR1 has also been confirmed in those conditions.[5,11] 5αR-expression was studied by other groups as well resembling the results that 5αR1 is predominantly present in the epithelium whereas 5αR2 is found in mesenchymal cells.[12] Furthermore, they showed that in contrast to 5αR2, 5αR1-expression was androgen-independent. Perhaps one speculation about such different findings is that technical differences and limited specimens may be responsible for such opposite findings.

Fetal formation of parts of the male urogenital tract and the development of androgen-responsive tissues during subsequent life are induced directly by testosterone and DHT as a downstream product catalyzed through 5αR-activity.[13] Androgen receptors bind DHT with higher affinity such activating gene-transcription at lower concentrations than testosterone.[14] Initiation of prostate differentiation as well as seminal vesicle sprouting coincide with incipient 5αR-production between Weeks 9 and 10. DHT also mediates penile and urethral development during Weeks 12 to 14 while disruptions of that pathway are supposed to cause hypospadias.[9] Transcriptional levels of 5αR are maximum between the second and third trimester decreasing with continuing pregnancy. Testosterone levels are concomitantly increasing further enhancing 5αR2-expression.[12,15] Concurrently, prostatic development accelerates excessively and bladder-prostate size ratio is counterbalanced in Week 24.[15] Rising quantities of epithelial buds and stroma come into sight from Week 22. Fascinatingly, DHT levels in the prostate remain normal with ageing regardless of declining plasma testosterone.[16] This inconsistency may take place because prostatic steroidogenic enzymes such as 17 βHSD are competent in synthesizing androgens from adrenal-derived dehydroepiandrosterone. These enzymes are expressed in response to androgens. A number of subtypes are anabolic while others are degrading hormones. 17 βHSD 2 inactivates testosterone to 4-dione at the same time as 17 βHSD 3 catalyzes the identical reaction in an opposite direction. 17 βHSD 7 metabolizes DHT to 3β-diol and transforms estrone to estradiol, being a key molecule modulating the estrogen-androgen balance.[2] These clashing findings emphasize the complexity of prostate development almost certainly requiring 5αR and additional local control devices like 17 βHSD.

## INFLUENCE OF 5αR1 AND 5αR2 ON THE FETAL DEVELOPMENT OF THE EXTERNAL GENITALIA

**Figure 2:**
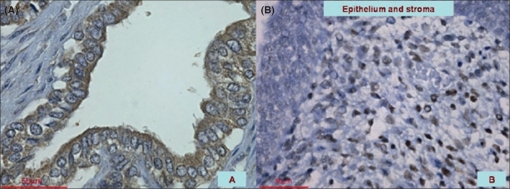
(A and B) *Expression of 5αR1 and 5αR2 at the end of gestation and postnatally (magnification 400×)*: (A) At the end of gestation 5αR1 is particularly accumulated within the epithelium with a small number of positively stained cells in the stroma; (B) Postnatal at the age of five months stromal staining can be clearly detected for 5αR2 being more intense than for 5αR1

5αR1 and 5αR2 have been found to be expressed in almost all tissues including the prostate, although the role of 5αR1 remains unclear.[3,17] Lack or malfunction of 5αR2 results in the clinical syndrome of 5αR-deficiency causing undervirilized males. Most affected subjects have genital ambiguity with a rudimentary prostate being often raised as females.[18,19] Differentiation of the testosterone-dependent mesonephric duct is obviously normal in these patients. Virilization occurs during puberty, frequently associated with a gender change. 5αR1 is normal in these patients as well. This entity stands for a clinical model of testosterone and DHT effects involved in sex differentiation. Their prostates are frequently impalpable and sonographic prostate volumes much smaller than in age-matched controls. Even though secondary virilization occurs there is little glandular tissue and the PSA is usually undetectable. Peripherally located 5αR1 is responsible for virilization producing adequate levels of DHT.[10,19,20] These observations could imply that exclusive 5αR1 may not be sufficient for adequate fetal prostate development. At the moment it seems to be more appropriate to assume that both isoforms are mandatory to complete organ differentiation and growth [Figure 2A, B]. Nonetheless, it appears puzzling that mice with 5αR1- and 5αR2-deficiency develop normal genitalia suggesting the presence of redundant mechanisms.[19]

## POSSIBLE ROLE OF 5αR1 AND 5αR2 ON THE EXPANSION OF PROSTATE CANCER

The higher fetal 5αR-expression in the epithelium than in the stroma is remarkable with respect to the expansion of prostate cancer (PCa) which also develops in the epithelial part of the gland. These observations are corroborated by findings of other groups showing increased 5αR1-activity but with declining 5αR2-activity during PCa development. As a result it may be concluded that both isoenzymens may participate in the pathophysiology of PCa.[21] 5αR1 has been detected in regions of budding and branching[8] implying participation in epithelial-mesenchymal interactions, and cancer cells use comparable mechanisms to assault underlying tissues. Consequently, issues come up about similarities between PCa and fetal development.[5,21]

It is well accepted that androgens may influence PCa development. Experiments assumed that cutting androgen levels with 5αR2 inhibitors might diminish prostate cancer risk.[22] But interestingly, while a reduction of cancer incidence was shown, those diagnosed displayed quite aggressive behavior.[17] Although the task of 5αR1 in this phenomenon remains unidentified one may guess that this alternative pathway is involved in high-grade PCa development suggesting that inhibition of 5αR2 may facilitate 5αR1 action in target tissues.[5,21] Similar 5αR-expression patterns in fetuses and PCa could symbolize differentiation processes already starting with fetal prostate tissue imprinting.[5,8,21,23]

## CONCLUSION

During fetal development of human prostates as well as during infancy both isoenzyme forms of 5αR are expressed. Contrary to previous studies it seems that disturbances of the 5αR2-pathway alone are not the only factors involved in the pathophysiology of developing genital organs as well as in prostate cancer formation. 5αR1 and other aspects like 17-βHSD may participate in physiological and pathological proceedings. Even though 5αR1 may be widely abundant in the developing prostate its biological significance in the process of organ differentiation, gland formation, and induction or progression of cancer remains unclear. However, its relatively distinct expression throughout all gestational stages may challenge the present understanding of 5αR1-metabolism in the human prostate warranting further research.
